# Dataset on broadband electrochemical impedance spectroscopy of Lithium-Ion batteries for different values of the state-of-charge

**DOI:** 10.1016/j.dib.2022.108589

**Published:** 2022-09-11

**Authors:** Emanuele Buchicchio, Alessio De Angelis, Francesco Santoni, Paolo Carbone, Francesco Bianconi, Fabrizio Smeraldi

**Affiliations:** a Department of Engineering, University of Perugia, Italy; b School of Electronic Engineering and Computer Science, Queen Mary University of London, UK

**Keywords:** Batteryelectrochemical impedance spectroscopy, state of charge estimation, Broadband electrochemical impedance spectroscopy, Multisine excitation, SOC, State Of Charge, EIS, Electrochemical Impedance Spectroscopy

## Abstract

This dataset consists of electrochemical impedance spectroscopy measurements on commonly-used batteries, namely Samsung ICR18650-26J cylindrical Lithium-Ion cells. The complex impedance of the batteries was measured at a set of fourteen different frequencies from 0.05 Hz to 1000 Hz, using a random-phase multi-sine excitation signal. For each excited frequency, the current amplitude was 50 mA, resulting in a measurement uncertainty of approximately 0.1 mΩ.

Six measurement repetitions are provided at ten different states-of-charge of four different brand-new batteries. Repeated EIS measurement results were obtained, for each individual battery cell, from six separate discharge cycles. All measurements were performed with the battery placed in a temperature-controlled chamber at 25 ± 1 °C. Batteries were allowed to thermalize before each measurement.

**Specifications Table**

**Table:** 

Subject:	Energy Engineering and Power Technology
Specific subject area:	Electrochemical impedance spectroscopy (EIS) of rechargeable lithium batteries, measured at different states-of-charge
Type of data:	Table
How the data were acquired:	The EIS data were obtained by measuring the complex impedance of Samsung ICR18650-26J cylindrical Lithium-Ion batteries at a set of different frequencies. The complex impedance measurement was performed using custom-made instrumentation, which consist of an in-house built electronic circuit (implementing a voltage-to-current converter, shunt resistor, and instrumentation amplifiers) and a commercial data acquisition board (Keysight U2351A).
Data format:	Raw
Description of data collection:	The EIS data, i.e. the imaginary and real parts of the battery impedance, were collected at the following frequencies [Hz]: 0.05, 0.1, 0.2, 0.4, 1, 2, 4, 10, 20, 40, 100, 200, 400, 1000. The EIS spectrum was measured at the following values of the state-of-charge (SOC): 100%, 90%, 80%, 70%, 60%, 50%, 40%, 30%, 20%, and 10%. The measurement was repeated six times on separate discharge cycles of each of four Samsung ICR18650-26J, 3.7 V, 2600 mAh Lithium-ion batteries. The measurements were performed with the battery placed in a temperature-controlled chamber, at a temperature of 25 ± 1 °C. Batteries were allowed to thermalize before each measurement.
Data source location:	• Institution: University of Perugia, Department of Engineering • City: Perugia • Country: Italy • Latitude and longitude: 43.1187°, 12.3574°
Data accessibility:	Repository name: Dataset on broadband Electrochemical Impedance Spectroscopy of Lithium-Ion Batteries for Different Values of the State-of-Charge Data identification number: 10.17632/mbv3bx847g.3 Direct URL to data: https://data.mendeley.com/datasets/mbv3bx847g

## Value of the Data

• This dataset [1] consists of original data that has not previously appeared in any other paper or data repository. In particular, the data consist of EIS measurement results on commonly-used batteries, performed at different state-of-charge values. The dataset contains repeated EIS measurement results obtained through different charging cycles on individual battery cells. Batches of measurements performed on different cells of the same manufacturer and model are included in the dataset.
• These data can be used to study the frequency-domain characteristics of batteries. Moreover, they can be employed to develop estimation methods, and for training machine learning models, aiming at the design of diagnostic tools for safe and efficient operation of battery-powered systems.
• Researchers in the battery, electrochemistry, power sources, and energy storage fields can benefit from these data. Furthermore, they can be useful for research and development engineers in the consumer electronics, power systems, and electric transportation fields.

## 1 Data Description

**Fig. 1:**
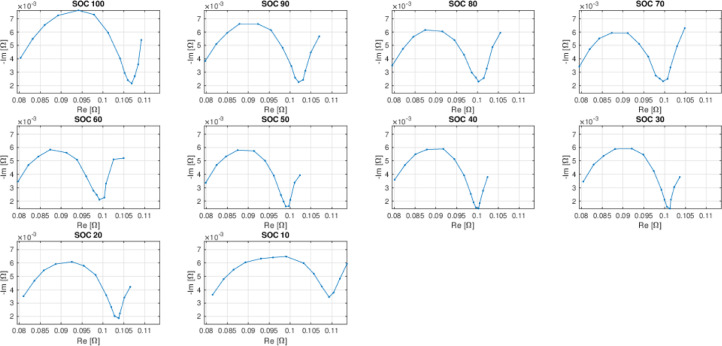
An example of Cole-Cole plot representations of EIS measurement data for a battery belonging to the dataset.

The dataset consists of two CSV files containing data from electrochemical impedance spectroscopy (EIS) of rechargeable lithium batteries (Fig. 1), measured at different states-of-charge (SOC) on four individual Samsung ICR18650-26J model batteries. All EIS spectra were computed measuring impedance at frequencies [0.05, 0.1, 0.2, 0.4, 1, 2, 4, 10, 20, 40, 100, 200, 400, 1000] Hz.

### 1.1 Impedance Data File

All measured impedance values are collected in a single CSV data file named *impedance.csv*. The file lists one complex impedance measurement per row. Each row includes the fields described in Table 1.

**Table 1:** Fields in impedance data file rows.

Field name	Description
MEASURE_ID	the unique identification code of each EIS measurement
SOC	state-of-charge of the battery
BATTERY_ID	the unique identification code of the battery
FREQUENCY_ID	key to the frequency values listed in frequencies.csv (labeled in increasing order)
IMPEDANCE_VALUE	measured complex impedance value in Ohm expressed in Cartesian form

### 1.2 Frequencies Data File

The *frequencies.csv* file contains the lookup table needed to retrieve the frequency value in Hertz from the *frequency_id* field in the *impedence.csv* data rows.

## 2 Experimental Design, Materials and Methods

**Fig. 2:**
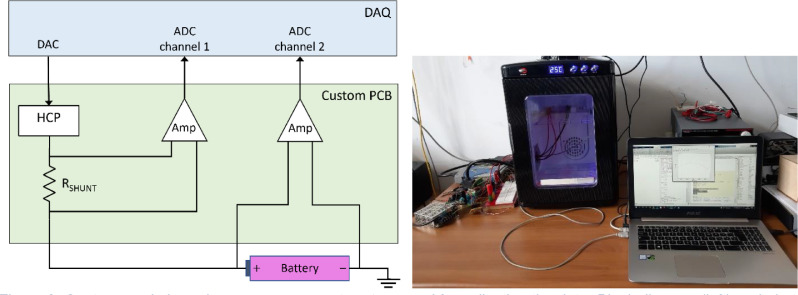
Custom-made impedance measurement system used for collecting the data. Block diagram (left) and photo of the experimental setup (right).

The data were obtained using a custom-made impedance measurement system, which implements a four-wire connection to the battery under test. The architecture of the system is illustrated by the diagram in Fig. 2, together with a picture of the experimental setup. A detailed description is available in [2]. Specifically, a data acquisition board (DAQ), Keysight U2351A, is used to provide the excitation signal by means of a 16-bit digital to analog converter (DAC) and to acquire the current and voltage signals via two 16-bit analog to digital converter (ADC) channels.

**Fig. 3:**
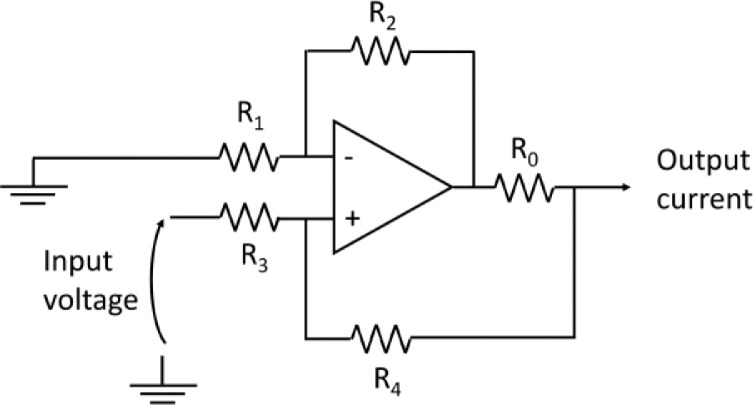
Schematic of the Howland current pump (HCP) circuit, which is used to convert a voltage signal to a current signal for exciting the battery under test.

The excitation signal generated by the DAC is converted to a current signal by a Howland current pump (HCP) circuit that acts as a voltage-to-current converter [3]. The schematic of the HCP circuit is shown in Fig. 3. To implement the HCP circuit, a power operational amplifier is used, namely Texas Instruments OPA541. The resistor values used in the HCP circuit are R0 = 1 Ω, R1 = R3 = 10 kΩ, R2 = R4 = 1 kΩ, resulting in a gain of 1/10.

The current obtained at the output of the HCP is then fed to the battery under test and measured by a 0.2 Ω, 1% tolerance, shunt resistor connected to an instrumentation amplifier (INA), namely Texas Instruments INA186, with a gain of 25, an offset of 2.5 V, and an output range of 0–5 V. The output of the INA is connected to the first ADC channel of the DAQ.

The voltage across the battery is also measured by a second INA, Analog Devices AD8421, with unity gain and offset compensation, which is connected to the second ADC channel of the DAQ. The HCP, the two INAs, and the shunt resistor are mounted on a custom-designed printed circuit board. Each of the batteries under test is a Samsung ICR18650-26J cylindrical Lithium-Ion cell, with a nominal voltage of 3.7 V and 2600 mAh nominal capacity. The battery is placed inside a temperature-controlled chamber where the temperature is 25 ± 1 °C.

In order to measure the impedance, the signals acquired by the ADC channels with a sampling rate of 10 kSample/s are transferred to a PC. There, the discrete Fourier Transform (DFT) is computed, and the complex impedance is obtained at each excited frequency by dividing the DFT of the voltage by that of the current.

The excitation signal is a random-phase multisine, i.e. the sum of harmonically-related sinusoids [4]. This type of broadband excitation signal allows to simultaneously excite the battery at a wide range of frequencies and perform EIS in a shorter time compared to the single sine approach. Specifically, a complete EIS measurement using a multisine excitation requires a time interval of length *T* = 40 s. Conversely, the single sine approach, where each sinusoid is measured separately for the same amount of time, would require a time interval of length NT, where N is the number of sinusoids. In our case, the system employs a quasi-logarithmic frequency spacing, where the excited frequencies are 0.05, 0.1, 0.2, 0.4, 1, 2, 4, 10, 20, 40, 100, 200, 400, and 1000 Hz. The excited frequencies, sampling rate, and number of samples are selected to ensure coherent sampling, where the acquired data record contains an integer number of periods of each sinusoidal component of the multisine. This allows for the elimination of spectral leakage in computation of the DFT. The current amplitude of each excited frequency component of the multisine is 50 mA, which results in a measurement uncertainty of approximately 0.1 mΩ, as characterized in [2].

To measure EIS at different values of SOC, the battery was first fully charged according to the manufacturer's specifications. Then, the EIS was measured at SOC 100%. After the measurement, the battery was discharged at a constant current of 1 A for 936 s, thus removing an amount of charge that corresponds to 10% of the nominal capacity. Subsequently, after a relaxation period of 1800 s, the EIS measurement was repeated. This cycle was iterated until a SOC level of 10% was reached.

## Ethics Statements

No human subjects or animals were involved in this works. No data was collected from social media platforms.

## CRediT authorship contribution statement

**Emanuele Buchicchio:** Conceptualization, Methodology, Software, Data curation, Writing – original draft, Writing – review & editing. **Alessio De Angelis:** Conceptualization, Methodology, Software, Data curation, Writing – original draft, Writing – review & editing. **Francesco Santoni:** Conceptualization, Methodology, Software, Data curation, Writing – original draft, Writing – review & editing. **Paolo Carbone:** Conceptualization, Methodology, Writing – review & editing, Supervision. **Francesco Bianconi:** Data curation, Writing – review & editing. **Fabrizio Smeraldi:** Data curation, Writing – review & editing.

## Declaration of Competing Interest

The authors declare that they have no known competing financial interests or personal relationships that could have appeared to influence the work reported in this paper.

## Data Availability

Dataset on broadband Electrochemical Impedance Spectroscopy of Lithium-Ion Batteries for Different Values of the State of Charge (Original data) (Mendeley Data).
